# Application of Artificial Intelligence Models in Oral and Maxillofacial Prosthesis Restoration: A Systematic Review

**DOI:** 10.1016/j.identj.2025.103861

**Published:** 2025-09-12

**Authors:** Rui Luo, Tengteng Zhang, Guo Lin, Qihan Tang, Zelin Fu, Lujin Cheng

**Affiliations:** a Xinjiang Medical University Stomatological College, Urumqi, China; b Department of dental prosthetics and Implants, the First Affiliated Hospital of Xinjiang Medical University, Urumqi, China; c Oral Disease Institute of Xinjiang Uyghur Autonomous Region, Urumqi, China

**Keywords:** Artificial intelligence, Maxillofacial prosthesis, Image acquisition, Data analysis, Prosthesis colouring

## Abstract

**Background:**

Artificial intelligence (AI) is increasingly favoured in the medical field, and the traditional maxillofacial prosthetic system has complex processes, low efficiency, and relatively insufficient model matching and aesthetic outcomes. With the rapid development of AI, it is expected that AI technology will address the shortcomings of these traditional methods.

**Purpose:**

To comprehensively analyse the research on AI in maxillofacial prosthetic image acquisition, data analysis, and colouring, and to elaborate on its application evaluation and future trends to improve the diagnosis and treatment experience of patients.

**Material and methods:**

Based on the PICO principle, we searched the PubMed, Web of Science, and Cochrane databases, as well as conducted manual searches, and screened English articles from January 2016 to January 2024 according to specific inclusion and exclusion criteria. Quality assessment was performed by 2 investigators using the JBI Quasi-Experimental Study Critical Assessment Checklist.

**Results:**

A total of 17 related articles were identified, including 7 in the field of image acquisition, 8 in the field of data analysis, and 2 in the field of pseudo colouring. The results show that the application of AI in these 3 fields is on the rise, reaching a record high in 2024.

**Conclusions and Clinical Relevance:**

There is a lack of literature available for analysis, all studies have demonstrated high accuracy and clinical necessity. However, there is a relative lack of consideration when it comes to the comfort and efficiency of patients and doctors in practice, which is a key direction for future AI software development. In addition, we need more research on irregular bone or personalized model design, as well as more accurate analysis of complex structures that are vulnerable to damage.

## Introduction

Craniofacial defects may result from trauma, congenital deformities, infectious processes, or tumour treatment.^1^ Such facial defects often lead to severe physical and emotional problems for patients. For example, mandibular defects caused by radiation-induced osteonecrosis account for 5% to 15% of patients who have undergone radiotherapy to the head and neck.^2^ Orofacial clefts, the most common congenital maxillofacial deformities, have seen a global prevalence increase of 40.3% in 2021 compared to 1990.^3^ These statistics highlight the severity of maxillofacial defects. For these patients, maxillofacial prosthetic surgery to replace missing structures can not only restore facial function but also significantly improve facial aesthetics^4^ and quality of life.^5^ As the demand for maxillofacial rehabilitation increases, the precision and fit of prostheses are becoming more critical. Although prostheses offer superior aesthetic outcomes compared to surgical reconstruction,^1^ the production of prostheses is a challenging process that requires considering many complex factors to customize methods for each patient. However, the traditional design and production of maxillofacial prostheses still have many shortcomings, such as low production efficiency and an inability to meet patients’ aesthetic needs fully.

It is well known that in the medical field, artificial intelligence (AI) has demonstrated capabilities that surpass human abilities. It can improve the accuracy of medical procedures, reduce costs, save time, and minimize human errors to the greatest extent.^6^, ^7^, ^8^ As a decision-making tool for diagnosis, treatment planning, and disease prediction, AI is becoming increasingly popular in the medical field.^9^, ^10^, ^11^, ^12^, ^13^ AI-driven models, such as machine learning, deep learning,^14^ and neural networks,^15^ have shown high precision in radiological analysis, disease detection, restorative design, and personalized design.^16^ Given the complexity of maxillofacial prostheses^17^ and the various patient-related factors that need to be considered during treatment, AI algorithms hold the potential to analyse patient data and design and manufacture maxillofacial prostheses more quickly based on individual patient needs and anatomical considerations. This application can enhance the precision and personalization of maxillofacial prostheses, ultimately improving and optimizing patient outcomes.^8^^,^^18^, ^19^, ^20^

Currently, the application of AI in maxillofacial prosthetic rehabilitation includes a series of processes such as image acquisition, data analysis, prosthesis design, colouring, and postoperative evaluation. We have observed an increasing trend in the number of AI-related articles published in the fields of image acquisition, data analysis, and prosthetic colouring in recent years. Research on improving and innovating AI models is also growing, demonstrating the potential for further development in these 3 areas. Despite this, research on AI in maxillofacial prosthetics is still in its infancy. However, its applications have already yielded some positive results and revealed current challenges. Therefore, the achievements of relevant studies not only need to be systematically summarized, but the issues and suggestions they raise also need to be refined and analysed to provide valuable insights for future research directions. Against this backdrop, the purpose of this article is to comprehensively review the research on AI in image acquisition, data analysis, and colouring related to maxillofacial prosthetics. We aim to evaluate the application of AI in these aspects of maxillofacial prosthetics and explore the potential future trends in each area, leveraging the power of AI to address the inefficiencies, underfitting, and poor aesthetics associated with traditional prosthesis production, in order to provide patients with a better treatment experience.

## Material and methods

This systematic review was not registered. This systematic review conforms to the Preferred Reporting Items for Systematic Reviews and Meta-Analyses (PRISMA) guidelines.^21^

### PICO strategy

The PICO strategy was used for the search: Population (P) includes patients who received fixed and mobile prosthesis treatment; Intervention (I) includes the application of different types of AI; Comparison (C) includes image acquisition technique, data analysis, and prosthesis colouring; Outcome (O) includes the practical application effect.

### Search strategy

Two researchers (Luo Rui and Zhang Tengteng) accessed 3 databases: PubMed, Web of Science, and Cochrane. Articles published in English between January 2016 and November 2024 were selected. Table 1 describes the detailed search strategies for each database. After the search was completed, EndNote (Version 21.4) was used to screen the final articles according to the inclusion and exclusion criteria.

**Table 1:** Search Strategy (WPS Office [Version 11.1.0.13703, Kingsoft, 2023]).

Database	Number of hits	Search string
PubMed	92	((Maxillofacial Prostheses) OR (Prostheses, Maxillofacial) OR (Maxillofacial Prosthesis) OR (Prosthesis, Maxillofacial) OR (Maxillary Prosthesis) OR (Maxillary Prostheses) OR (Prostheses, Maxillary) OR (Prosthesis, Maxillary)) AND ((Artificial Intelligence) OR (Intelligence, Artificial) OR (Computer Reasoning) OR (Reasoning, Computer) OR (AI (Artificial Intelligence)) OR (Machine Intelligence) OR (Intelligence, Machine) OR (Computational Intelligence) OR (Intelligence, Computational) OR (Computer Vision Systems) OR (Computer Vision System) OR (System, Computer Vision) OR (Systems, Computer Vision) OR (Vision System, Computer) OR (Vision Systems, Computer) OR (Knowledge Acquisition (Computer)) OR (Acquisition, Knowledge (Computer)) OR (Knowledge Representation (Computer)) OR (Knowledge Representations (Computer)) OR (Representation, Knowledge (Computer)))
Web of Science	74	TS=((Maxillofacial Prostheses) Or (Prostheses, Maxillofacial) Or (Maxillofacial Prosthesis) Or (Prosthesis, Maxillofacial) Or (Maxillary Prosthesis) Or (Maxillary Prostheses) Or (Prostheses, Maxillary) Or (Prosthesis, Maxillary)) And ((Artificial Intelligence) Or (Intelligence, Artificial) Or (Computer Reasoning) Or (Reasoning, Computer) Or (AI (Artificial Intelligence)) Or (Machine Intelligence) Or (Intelligence, Machine) Or (Computational Intelligence) Or (Intelligence, Computational) Or (Computer Vision Systems) Or (Computer Vision System) Or (System, Computer Vision) Or (Systems, Computer Vision) Or (Vision System, Computer) Or (Vision Systems, Computer) Or (Knowledge Acquisition (Computer)) Or (Acquisition, Knowledge (Computer)) Or (Knowledge Representation (Computer)) Or (Knowledge Representations (Computer)) Or (Representation, Knowledge (Computer)))
Cochrane	2	((Maxillofacial Prostheses*) OR (Prostheses, Maxillofacial*) OR (Maxillofacial Prosthesis*) OR (Prosthesis, Maxillofacial*) OR (Maxillary Prosthesis*) OR (Maxillary Prostheses*) OR (Prostheses, Maxillary*) OR (Prosthesis, Maxillary*)) AND ((Artificial Intelligence*) OR (Intelligence, Artificial*) OR (Computer Reasoning*) OR (Reasoning, Computer*) OR (AI (Artificial Intelligence)*) OR (Machine Intelligence*) OR (Intelligence, Machine*) OR (Computational Intelligence*) OR (Intelligence, Computational*) OR (Computer Vision Systems*) OR (Computer Vision System*) OR (System, Computer Vision*) OR (Systems, Computer Vision*) OR (Vision System, Computer*) OR (Vision Systems, Computer*) OR (Knowledge Acquisition (Computer)*) OR (Acquisition, Knowledge (Computer)*) OR (Knowledge Representation (Computer)*) OR (Knowledge Representations (Computer)*) OR (Representation, Knowledge (Computer)*))

### Inclusion criteria

Articles related to the application of AI in the field of image acquisition, data analysis, and pseudoprosthesis colouring in maxillofacial prosthesis, among which the types of maxillofacial prosthesis include jaw prosthesis, nose prosthesis, eye prosthesis, ear prosthesis, etc. Included articles published in English between January 2016 and November 2024.

### Exclusion criteria

1) Articles that are not relevant to AI research after initial inclusion;
2) Articles on AI research related to other disciplines and unrelated to maxillofacial prosthesis;
3) Studies related to maxillofacial prosthesis but unrelated to AI;
4) Studies related to the maxillofacial region and AI but unrelated to prosthesis;
5) Articles of review literature, case reports, review articles, and letters to the editor.

Two researchers (Rui Luo and Tengteng Zhang) collected the filtered articles into a table. The quality of the studies was assessed independently by applying the list of critical assessments for quasi-experimental studies (non-randomised experimental studies) proposed by the Joanna Briggs Institute (JBI) Centre for Evidence-based Health Care Research in Australia (Table 2).

**Table 2:** Joanna Briggs Institute (JBI) list of key assessments for quasi-experimental studies (non-randomized experimental studies).

Question	Answer
1. Is it clear in the study what the cause is and what the ‘effect’ is (that is, there is no confusion about which variable comes first)?	Yes, No, Unclear, or Not applicable.
2. Were the participants included in any similar comparisons?	
3. Were the participants included in any comparisons receiving similar treatment/care other than the exposure or intervention of interest?	
4. Was there a control group?	
5. Were there multiple measurements of the outcome, both before and after the intervention/exposure?	
6. Was the follow-up complete, and if not, were the differences between groups in terms of their follow-up adequately described and analysed?	
7. Were the outcomes of participants included in any comparisons measured in the same way?	
8. Were outcomes measured reliably?	
9. Was an appropriate statistical analysis used?	

## Results

**Fig. 1:**
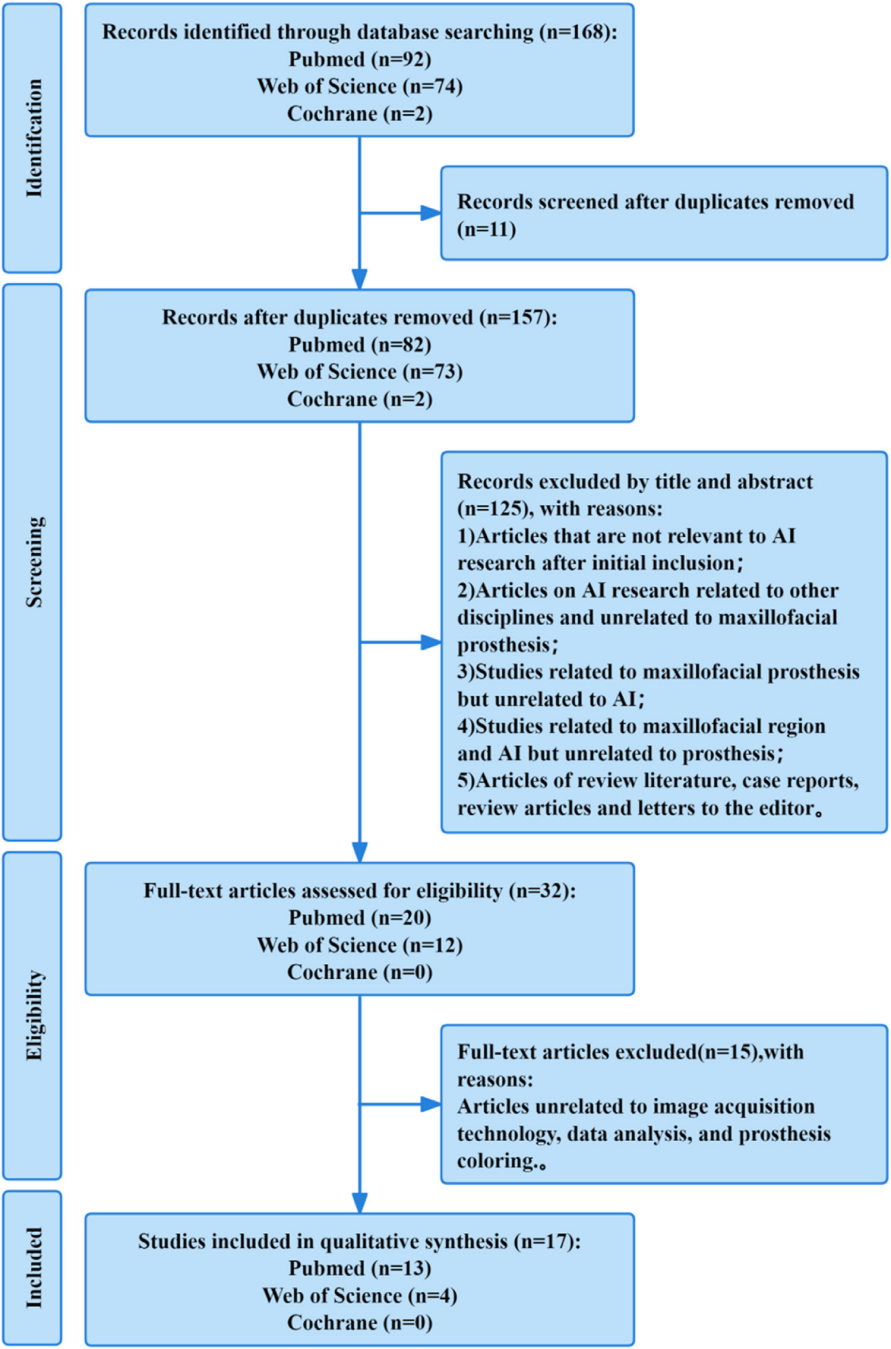
PRISMA flowchart. (WPS office [version 11.1.0.13703, Kingsoft, 2023]).

Based on the predefined search strategy, we conducted an initial search in 3 databases – PubMed, Web of Science, and Cochrane. This preliminary search has identified 168 studies published between January 2016 and November 2024. After screening the titles and abstracts and conducting a rationality analysis, a total of 17 articles were included for systematic review. Among these studies, 7 were in the field of image acquisition, 8 in the field of data analysis, and 2 in the field of prosthesis colouring (Figure 1).

**Fig. 2:**
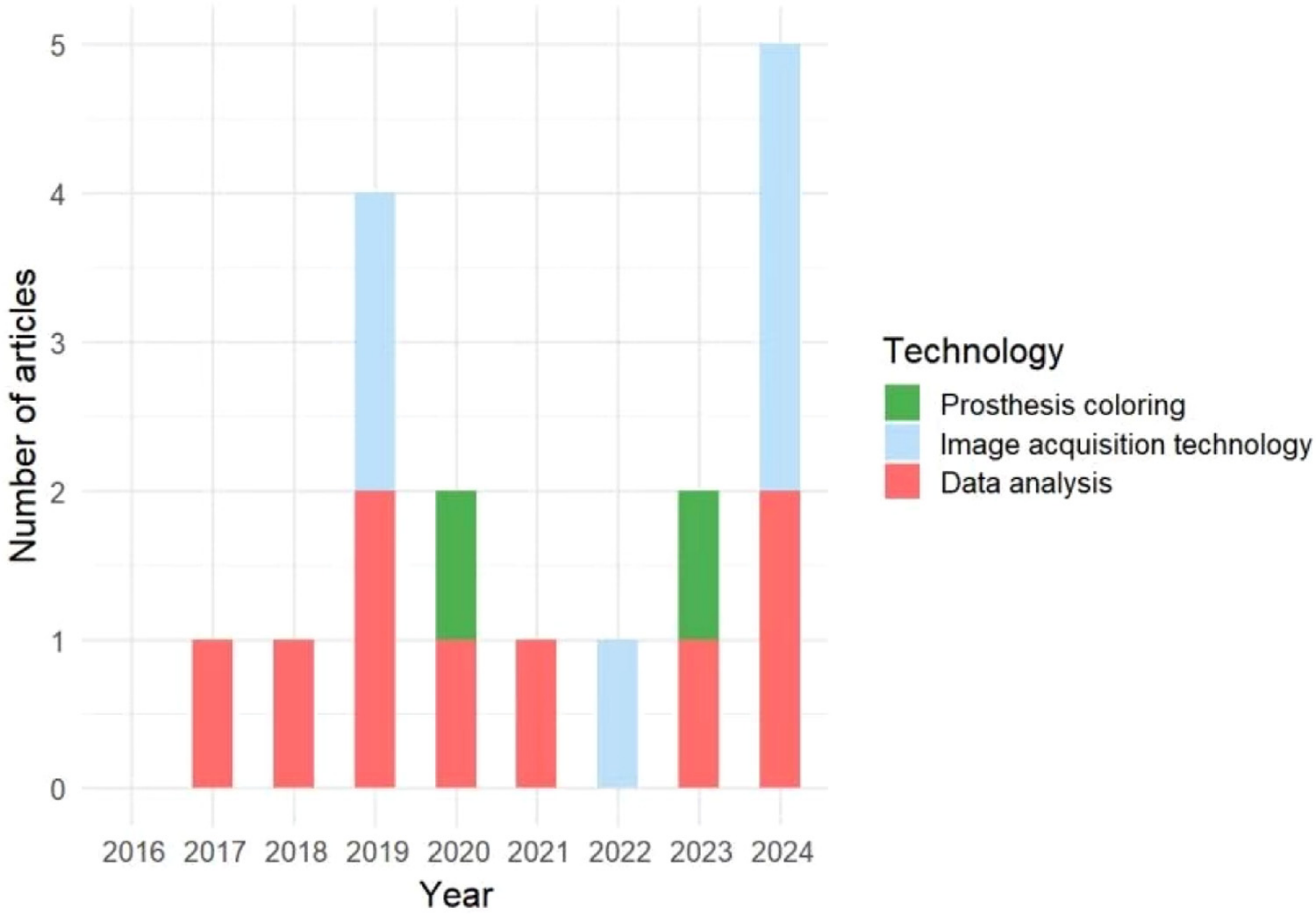
Number of included articles (generated with R version 4.4.3, ggplot2 package).

Our search aimed to cover research articles on AI in the fields of image acquisition, data analysis, and prosthetic colouring published between January 2016 and November 2024. Figure 2 shows that the application of AI in this field has been on an upward trend since 2016, reaching a record high in 2024. According to Poisson regression analysis (R version 4.4.3), the number of annual publications related to AI in maxillofacial prosthetics has shown an increasing trend (Incidence Rate Ratio [IRR] = 1.20, 90% CI: 1.02-1.42), with a positive correlation between year and number of articles (*P* = .066 < .10).

**Fig. 3:**
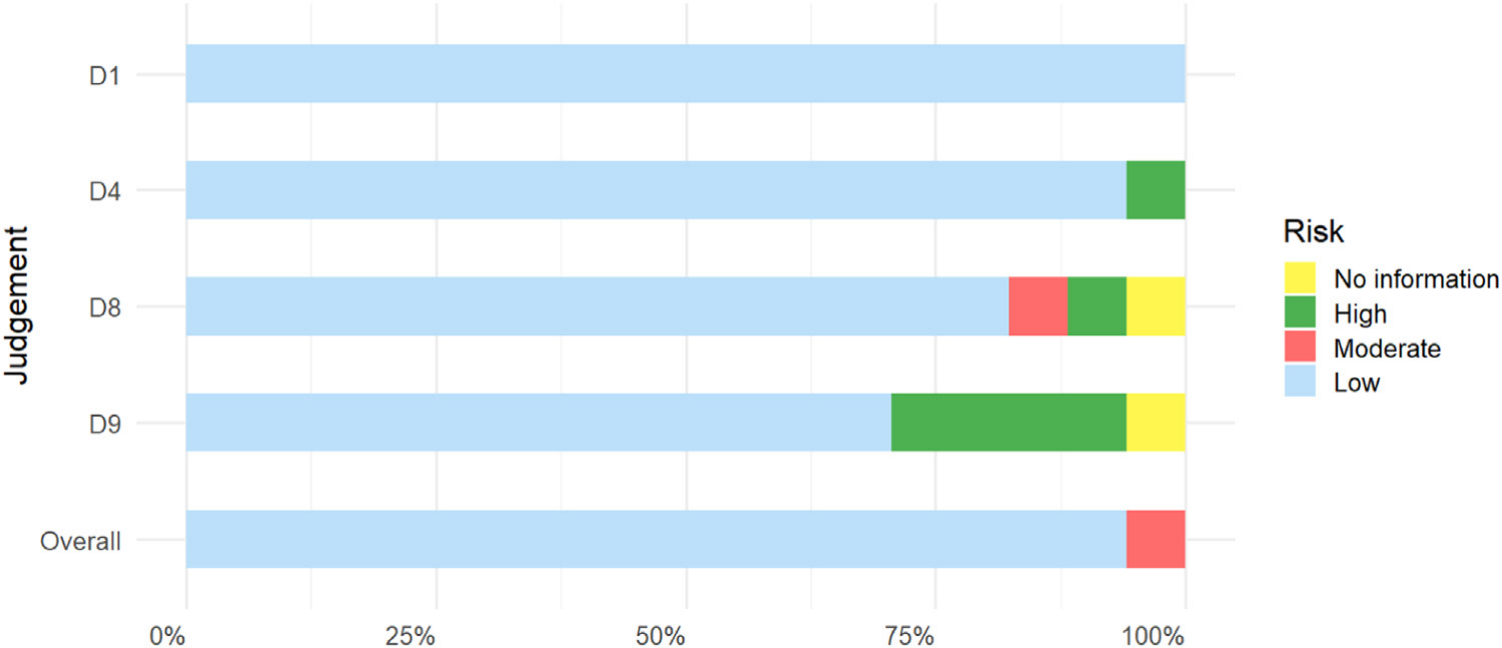
List of JBI evaluation results (generated with R version 4.4.3, ggplot2 package).

As this review study focuses on digital scan analysis, questions 2 and 6 of JBI do not apply to this systematic review. Questions 3, 5 and 7 do not apply to any included studies (Table 3). The key evaluation list of JBI quasi-experimental results showed that all included articles in question 1 had a low risk of bias. For question 4, all studies obtained a low risk of bias except Husain et al's study. As for question 8, all investigations reached a low risk of bias except for Gou's survey, which showed a high risk of bias, Husain's survey, which showed a moderate risk of bias, and Bellens' survey, which lacked information. As for question 9, all studies showed low risk of bias except Ferrini et al's survey, Beri et al's survey, Liu et al's survey, Husain et al's survey, and Bellens et al's survey, which lacked information. Overall, except for the study by Husain et al, which showed a moderate risk of bias, all studies showed a low risk of bias (Figure 3).

**Table 3:** JBI assessment results table (WPS office [version 11.1.0.13703, Kingsoft, 2023]).

Deviation risk						
	D1	D4	D8	D9	Overall	
Minnema et al^37^	**+**	**+**	**+**	**+**	**+**	
Li et al, 2021^45^	**+**	**+**	**+**	**+**	**+**	
Kilina et al, 2024^46^	**+**	**+**	**+**	**+**	**+**	
Oh et al, 2018^47^	**+**	**+**	**+**	**+**	**+**	
Gou et al, 2022^48^	**+**	**+**	**×**	**+**	**+**	
Ferrini et al^28^	**+**	**+**	**+**	**×**	**+**	
Bellens et al^30^	**+**	**+**	**༟**	**༟**	**+**	
Revilla-León et al^27^	**+**	**+**	**+**	**+**	**+**	Judgement
Beri et al^29^	**+**	**+**	**+**	**×**	**+**	D1:Q1
Minnema et al^33^	**+**	**+**	**+**	**+**	**+**	D4:Q4
Ali et al^32^	**+**	**+**	**+**	**+**	**+**	D8:Q8
Liu et al^40^	**+**	**+**	**+**	**×**	**+**	D9:Q9
Husain et al^38^	**+**	**×**	**-**	**×**	**-**	
Wang et al^41^	**+**	**+**	**+**	**+**	**+**	× high
Kois et al, 2023^49^	**+**	**+**	**+**	**+**	**+**	- unclear
Mine et al^44^	**+**	**+**	**+**	**+**	**+**	+ low
Kurt et al^43^	**+**	**+**	**+**	**+**	**+**	༟No information.

As shown in Figure 3, the assessment results of the non-D9 group literature are highly concentrated in 'low risk'. In contrast, the proportion of 'low risk' literature in the D9 group is significantly reduced, and the proportion of 'medium and high risk' literature is relatively increased. A chi-square test was conducted for the D9 issue, and the Fisher test result was (OR = 0.20, *P* = .024 < .05).

## Discussion

This systematic review demonstrates a significant upward trend in AI applications for maxillofacial prosthetics since 2016, with an estimated 20.3% annual increase in relevant publications (effect size). This trend may be attributed to the rise of interdisciplinary fields, where the application of AI in various industries has achieved remarkable results. Additionally, as people's living standards improve, the increasing demands for efficiency and precision in the fabrication of maxillofacial prostheses make the application of AI the best choice. While limited by the 9-year sample size and potential publication bias (eg, exclusion of conference abstracts and non-English literature), the exploratory nature of this analysis suggests the actual research activity may be underestimated. Future validation through multilingual database expansion and patent data inclusion is warranted. These findings not only highlight AI's untapped potential in maxillofacial prosthetics but also signal an emerging paradigm shift toward AI-driven design and manufacturing.

In addition, the chi-square test conducted for Question 9 based on Figure 3 shows that the proportion of low risk in Question 9 is significantly different from that in other groups, which indicates that current research is relatively deficient in statistical analysis. It is expected that future studies will more fully interpret research results from a statistical perspective, which may lead to more valuable insights.

The included literature, based on the criteria for inclusion and exclusion, was further analysed in 3 areas: image acquisition, data analysis, and prosthesis colouring.

### Image acquisition technology

Patient image acquisition serves as the foundation for the fabrication of maxillofacial prostheses, with efficiency and precision during the acquisition process being the primary concerns for researchers. As a widely used clinical tool, intraoral scanners (IOS) can directly capture the 3-dimensional morphology and coloured texture information^22^^,^^23^ of intraoral soft and hard tissue surfaces, making them a hotspot for integration with AI.^24^^,^^25^ Oh et al found that AI-assisted IOS impression scanning achieved the lowest root mean square error (RMSE) values for trueness and precision in the maxillary dental arch compared to other scanning techniques (eg, direct scanning and impression scanning), with the highest trueness for individual abutments.^26^ Revilla-León et al demonstrated that the IOS calibration and AI articulation subgroups significantly outperformed the non-calibrated subgroup in terms of trueness and precision for the maximum intercuspal position of the maxillomandibular relationship.^27^ Ferrini et al, focusing on marginal fit analysis, concluded that all tested IOS devices could produce prostheses with satisfactory marginal gap values, with an overall average of 53.45 ± 30.52 μm, all within the clinically acceptable threshold of 120 μm. These findings suggest that AI-assisted digital impressions using IOS may be a viable alternative to traditional analogue techniques.^28^

Although AI has improved the performance of IOS, all researchers face common challenges in software technology, including limitations in image stitching, file format conversion, and interference from verification and correction procedures. These issues compromise the accuracy of scanning results and hinder workflow efficiency. Additionally, Oh et al pointed out that deficient scan areas from IOS acquisition, the preparation designs used in experiments, and the limitations of existing impression materials may affect overall data accuracy. Addressing these complex problems will require further in vitro studies and clinical data, and their resolution could significantly enhance the quality of image scanning.

Among the studies included in this review, many researchers focused solely on using AI to improve scanning trueness and precision, with limited consideration for enhancing the patient experience. Furthermore, much of the research on advancing image acquisition technology relies on relatively expensive 3D scanners. The goal of technological advancement should be to improve patient comfort and satisfaction while reducing labour and costs. Beri et al proposed a novel approach by using high-quality cameras (Nikon D5300 DSLR and Google Pixel 2 XL) as alternatives to high-end scanners for capturing high-resolution images. Their study employed Autodesk Recap Photo as photogrammetric software to create 3D mesh models of each plaster model, utilizing 2D image slices converted into 3D visualization and volume rendering techniques for maxillofacial applications. The root mean square (RMS) of photogrammetric scans was nearly equivalent to that of CT-scanned models from a Comet 3D scanner, with model completeness exceeding 94% and reaching over 98% within a 2 mm range. This study confirmed that DSLR and 60p Google Pixel cameras could serve as cost-effective alternatives to expensive 3D scanners, though scanning of bone grooves and defect apexes remained insufficient.^29^ These results suggest a more economical and efficient approach, where cameras combined with AI visualization and rendering could reduce costs while improving patient experience.

Beyond the aspects of precision and patient experience in image acquisition technology, improving the efficiency of model algorithms is another promising direction for future development. A recent study generalized the iteration reconstruction framework into a Learned Experts’ Assessment-based Reconstruction Network (LEARN), proposing a method combining deep learning (DL) with XCT scanning, which yielded impressive results. Under 64-view and 128-view conditions, their model achieved improvements of 5.2 dB and 3.1 dB in peak signal-to-noise ratio (PSNR), respectively, with root mean square errors (RMSE) of 0.0093 and 0.0068 and structural similarity index measures (SSIM) of 0.9660 and 0.9790. This intelligent approach optimizes image quality and accelerates reconstruction speed, representing a remarkable advantage and offering a potential tool for enhancing maxillofacial scanning efficiency.^30^ Integrating LEARN into maxillofacial trauma management and prosthetic fabrication could improve diagnostic efficiency and accuracy, thereby enhancing treatment planning, decision-making, and patient outcomes, and promoting personalized therapeutic effects.

### Data analysis

Data analysis is a crucial component of AI-based evaluation of image acquisition results, as steps such as model segmentation, optimization, and reconstruction are key to the final fabrication of maxillofacial prostheses. The multi-layered structure of convolutional neural networks (CNNs) enables feature extraction from images during training and recognition of these features in new images, thereby performing specific tasks.^31^ Based on existing CNN models, Ali et al compared 4 pre-trained models (VGG16, Inception-ResNet-V2, DenseNet-201, and Xception) for classifying maxillary conditions. Identify 7 different surgical categories, including cleft palate, dentate maxillectomy, edentulous maxillectomy, etc. The study demonstrated that these CNNs could assist in workload allocation based on difficulty levels, develop automated diagnostic systems, and integrate essential design information.^32^

In terms of improving model quality and efficiency, Minnema et al trained a CNN specifically designed for skull segmentation using labelled CT scans. The resulting model exhibited high quality (mean deviation = 0.44 mm ± 0.36 mm) and performed exceptionally well on defect edges (mean deviation = 0.27 mm ± 0.29 mm). Clinically, Minnema et al found that the fully automated CNN could flexibly adapt to variations across different CT scanners and imaging protocols while significantly reducing the time and effort required for current CT image processing. It also enabled precise skull segmentation, making additive manufacturing (AM) structures more accessible for patients.^33^ In their discussion, Minnema et al emphasized that further evaluation is needed to assess CNN performance across multiple orthogonal planes (ie, axial, sagittal, and coronal) as well as on low-dose CT and cone-beam CT scans.

Model optimization and training are essential for advancing AI. Research related to CNN requires large amounts of training images, but due to privacy regulations and ethical considerations, the amount of clinically available data is often limited. The establishment of data-sharing platforms and infrastructure (eg, cloud computing) will require broader collaboration among dental research consortia.^34^^,^^35^ To address this issue, another study by Minnema et al proposed a mixed-scale dense CNN (MS-D Net). Experimental results showed that the segmentation performance of MS-D Net was comparable to state-of-the-art U-Net and ResNet CNN architectures, with mean absolute deviations (MAD) of 0.44 ± 0.13 mm, 0.43 ± 0.16 mm, and 0.40 ± 0.12 mm, respectively. Moreover, MS-D Net preserved more anatomical details in the generated STL models while using fewer trainable parameters (n = 45,756). Reducing the number of parameters is critical in clinical applications, as it mitigates overfitting risks and prevents errors^36^ in clinical outcomes caused by common deep learning issues (eg, vanishing gradients and local minima). Another interesting finding was that MS-D Net demonstrated a stronger ability to learn features of relatively rare structures in the training dataset, making it more suitable for real-world clinical segmentation purposes. Deep learning technologies offer an effective solution for mitigating metal artifact-induced errors in computer-assisted surgery, particularly in specific types of image analysis. However, the development of gold standard-based bone segmentation remains time-consuming.^37^

Although some progress has been made in the optimization and refinement of maxillofacial prostheses, research on personalized design remains relatively scarce. AI has been employed to address this limitation, particularly in the creation of patient-specific mandibular prostheses. Husain et al developed a procedure based on the Morphological Anatomical Feature (MAF) method, along with a parametric bone model generated by their application. They proposed an automated process for creating user-defined features (UDFs) in CAD software, allowing for patient-specific customization. From a clinician's perspective, they emphasized that this UDF should provide a more intuitive interface, enabling faster preoperative planning or simulation of surgical interventions.^38^ A better-fitting prosthesis and a more streamlined fabrication process are crucial directions for improving prosthetic quality, making refined and automated digital analysis a key pathway to enhancing prosthesis outcomes.

Beyond image analysis, patient-specific plates tailored to individual anatomical conditions can significantly improve the prognosis of maxillofacial prostheses and enhance patient satisfaction. The application of finite element analysis (FEA) in craniofacial reconstruction surgery has been well recognized, as FEA serves as a powerful tool for assessing the biomechanical properties of complex geometries.^39^ In the study by Liu et al, customized plates underwent topology optimization and design modifications based on FEA results. By integrating FEA with optimization algorithms or numerical methods, they achieved structures that met stiffness requirements while minimizing material usage under low or no stress conditions. The study confirmed that FEA is an effective, accurate, and non-invasive method for predicting various biomechanical parameters of the human mandible.^40^ Most importantly, the design approach for customized fixation systems can achieve better biomechanical performance and mechanical behaviour while providing a more stable environment for bone healing. Combined with additive manufacturing (AM) technology, topology optimization enables the rapid design and fabrication of patient-specific plates for mandibular fracture fixation, improving both surgical quality and efficiency in clinical practice.

Technological advancements are not limited to enhancing patient experience alone – for clinicians and manufacturers utilizing AI, the efficiency and usability of software during actual operations also significantly impact treatment quality. Wang et al developed the software EasyImplant, which introduces a prosthetic design framework for creating zygomatic implants in animals. Compared to commercial platforms like 3-Matic, EasyImplant offers higher efficiency, better integration, and greater user-friendliness. Clinicians only need to import a 3D model reconstructed from CT data, and through a few simple steps, they can obtain the final porous implant within 10 minutes.^41^ However, despite improved usability, clinicians still require a foundational understanding of geometric design principles and effective communication with engineers. This phenomenon highlights the need for future AI research in prosthetics to focus on simpler, faster, and more user-friendly workflows – or exact, personalized structural design.

### Prosthesis colouring

The most essential factor in the aesthetic effect of maxillofacial prostheses is the colour match of the prostheses to the patient's skin,^42^ making the right prostheses requires an efficient and convenient way to determine the appropriate amount of pigment mix. Still, there are a few options available on the market, all of which are either too expensive or too technical. Kurt et al compared the performance of attention-gated cycle unit (GRU) and artificial neural network (ANN) algorithms on the colouring of silica-gel maxillofacial prosthetics. The RMSE (0.029 ± 0.0152) and mean absolute error (MAE) values (0.045 ± 0.0235) of the ANN algorithm are significantly higher than those of the attention-based GRU model. Therefore, GRU provides better performance than the ANN algorithm in MAE and RMSE values.^43^ One of the advantages of this attention-based GRU model is that it can be run on a single computer with a standard central processing unit, thus improving the operability of the colouring system.

In addition, Mine et al found several clinical reports that presented a complete digital workflow and discussed the possibility of printing colour silicone prostheses directly. Mine et al compared 2 machine learning algorithms, namely ANN-based deep learning and the random forest algorithm, and found that the deep ANN method produced better results than the random forest algorithm in terms of ΔE00 values.^44^ Skin colour matching technology based on real-time deep learning will further provide more economical and convenient colouring support for maxillofacial prosthetics. To improve the authenticity of prostheses and enhance patient satisfaction, they plan to conduct further evaluations of the repeatability and feasibility of these direct printing systems.

## Limitation

This systematic review has several limitations. Given that the research field is still in its early stages, the number of relevant studies available is limited (only 17 articles were included in this review), which may reduce statistical power and limit the generalizability and external validity of the study findings. Additionally, heterogeneity exists among the included studies – for example, in terms of image acquisition techniques, involving various experimental subjects (eg, human specimens, artificial models, and patient models) and multiple variable analyses (eg, RMSE values, time, completeness, model deviation values). This heterogeneity exists and complicates the integration and comparison of results. Due to challenges in obtaining clinical data or ethical restrictions – particularly in studies on image acquisition techniques – most experiments relied on existing models, lacking rigorous clinical trial validation, which may compromise the reliability and reproducibility of the findings.

This review focused on 3 critical aspects of maxillofacial prosthesis fabrication: image acquisition, data analysis, and prosthesis colouring, which have preliminarily achieved integration with AI technologies. However, further research remains constrained by limitations of information techniques and calculation program, especially when handling irregular or fragile anatomical structures. Deeper exploration is still needed. Future efforts should prioritize expanding sample sizes, standardizing study designs, and enhancing clinical validation to facilitate the translation of this technology into routine clinical practice. Meanwhile, interdisciplinary collaboration should be strengthened, and scanning equipment and hardware conditions should be improved to drive continuous technological advancements.

## Conclusion

AI demonstrates significant advantages in 3 key aspects of maxillofacial prosthetics: image acquisition, data analysis, and prosthesis colouring. However, to achieve optimal clinical outcomes, closer collaboration between clinicians and AI experts remains essential. Moving forward, enhancing the technical capabilities of AI software – reducing physicians’ workload, improving patient satisfaction, and optimizing design workflows – will be pivotal directions for AI development in this field.

## Author contributions

Rui Luo contributed to conception and design, data analysis, and interpretation, drafted and critically revised the manuscript; Tengteng Zhang contributed to data acquisition, auxiliary revised the manuscript; Guo Lin, Qihan Tang, Zelin Fu, and Lujin Chen contributed to data analysis and management; Lujin Cheng contributed to conception and design, data analysis and interpretation, and critically revised the manuscript. All authors gave final approval and agree to be accountable for all aspects of the work.

## Conflict of interest

None disclosed.

## Appendix Supplementary materials

Image, application 1

Image, image 2
