# Supplementary figures and images for: Application of Artificial Intelligence Models in Oral and Maxillofacial Prosthesis Restoration: A Systematic Review
Source: Int Dent J. 2025 Sep 12;75(6):103861. doi: 10.1016/j.identj.2025.103861 (PMC12790079; doi:10.1016/j.identj.2025.103861)

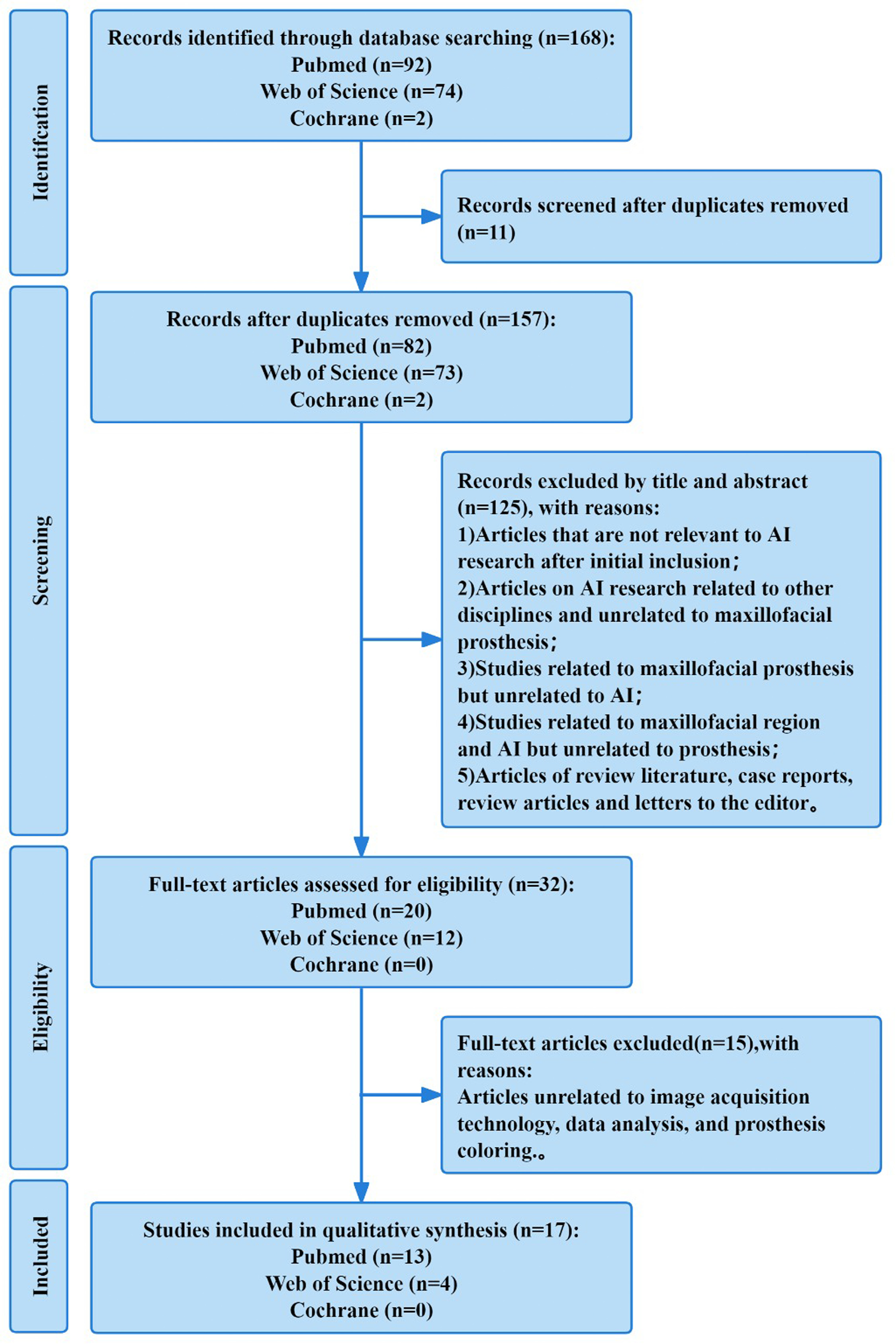

Supplement: Supplementary file 2 [file mmc2.jpg]
